# Late Complications After Previous Surgical Repair for Coarctation With Extra-Anatomic Bypass Graft: Report on Two Cases

**DOI:** 10.7759/cureus.12722

**Published:** 2021-01-15

**Authors:** Faisal Al-Husayni, Ahmed Samman, Mohammed Althobaiti, Abdulaziz Alghamdi, Wail Alkashkari

**Affiliations:** 1 Internal Medicine, King Abdullah International Medical Research Center, Jeddah, SAU; 2 Internal Medicine, National Guard Hospital, King Abdulaziz Medical City, Jeddah, SAU; 3 Cardiology, King Fahad General Hospital, Ministry of Health, Jeddah, SAU; 4 Radiology, King Abdullah International Medical Research Center, King Saud Bin Abdulaziz University for Health Sciences, Jeddah, SAU; 5 Radiology, King Abdulaziz Medical City, Ministry of National Guard Health Affairs, Jeddah, SAU; 6 Medicine, King Abdulaziz University, Jeddah, SAU; 7 Cardiology, King Abdullah International Medical Research Center, King Saud Bin Abdulaziz University for Health Sciences, Jeddah, SAU; 8 Cardiology, King Faisal Cardiac Center, King Abdulaziz Medical City, Ministry of National Guard Health Affairs, Jeddah, SAU

**Keywords:** coarctation of aorta, surgical repair, extra-anatomic bypass graft, late complications, anastomosis, aortic pseudoaneurysm, endovascular stent graft

## Abstract

Surgical correction of aortic coarctation (CoA) has been the standard treatment in infants and adolescents to prevent late consequences related to obstruction and distal hypoperfusion. Several surgical techniques for CoA repair have traditionally been applied. However, late complications, including re-CoA and pseudoaneurysm formation, are not uncommon. The incidence of complications is highly related to the type of initial surgery. Here, we are reporting two cases of late complications related to an infrequently used surgical technique, the extra-anatomical aortic bypass graft (EABG). The first case presented with pseudoaneurysm at the distal anastomosis site with the descending aorta and treated by endovascular stent graft. The second case presented with stenosis at the proximal anastomosis site with the left subclavian artery (LSCA) and treated medically upon the request of the patient.

## Introduction

Aortic coarctation (CoA) is defined as congenital stenosis of the aorta, most commonly located in the proximal descending aorta, just below the origin of the left subclavian artery (LSCA). CoA is the sixth most common congenital heart disease (CHD) accounting for 4%-8% of all CHD and occurs in four out of 1,000 live births with a male predominance [1]. It can occur as a solitary pathology limited to the aortic isthmus or can present as a more complex lesion, including long segment hypoplasia of the transverse aortic arch. The degree of the narrowing can range from a mild to severe form with interruption of the aorta at the site of the stenosis. CoA can occur as an isolated lesion but is often associated with other cardiovascular lesions, such as a bicuspid aortic valve (BAV) in 50%-75% of the cases, mitral valve abnormalities, subaortic stenosis, ventricular and atrial septal defects, aortic arch hypoplasia, and patent ductus arteriosus. The most important non-cardiac-associated lesion is a cerebral aneurysm, which is present in up to 10% of patients, which is approximately five times higher than that in the general population [2]. In infants and adolescents, surgical correction of CoA is considered the standard treatment to prevent early or late complications related to the obstruction. Untreated, the mortality and morbidity of CoA is high due to complications such as aortic dissection, infective endocarditis, severe aortic insufficiency, systemic hypertension, coronary artery disease, heart failure, and intracranial hemorrhage. Patients with CoA have a mean age of death of 34 years and 75% mortality by age 43 years if they survive beyond infancy [1-2].

Six surgical techniques for CoA repair have traditionally been applied: (1) end-to-end anastomosis, (2) extended end-to-end anastomosis, (3) patch aortoplasty, (4) subclavian artery flap aortoplasty, (5) interposition tube graft, and (6) extra-anatomical aortic bypass graft (EABG). Initial surgical repair of CoA yields excellent short-term results, however, long-term complications include aneurysm formation and re-CoA. The occurrence of both complications is dependent on the initial method for CoA repair, and they are not uncommon, which requires long-life surveillance of the patient [3].

EABG techniques aim to keep the stenosed aorta in place while providing adequate blood flow to the distal part of the aorta. This is achieved through a median sternotomy approach, with cardiopulmonary bypass support. The method of bypassing the CoA segment depends on an anastomosed to the ascending aorta or the subclavian artery proximally using a prosthetic conduit, which is anastomosed to the descending aorta distally. It is believed that EABG is the most practical and effective technique to repair CoA for adult patients, specifically in cases where interrupted aorta or hypoplastic aortic arch is present. Moreover, the usefulness of this technique magnitudes when another cardiac procedure such as coronary artery bypass surgery (CABG) or aortic valve replacement is needed [4].

We describe two cases of adults who underwent previous surgical repair for CoA with EABG and presented with two different late post-repair complications: aortic pseudoaneurysm and re-CoA at the anastomosis site.

## Case presentation

Case 1

**Figure 1:**
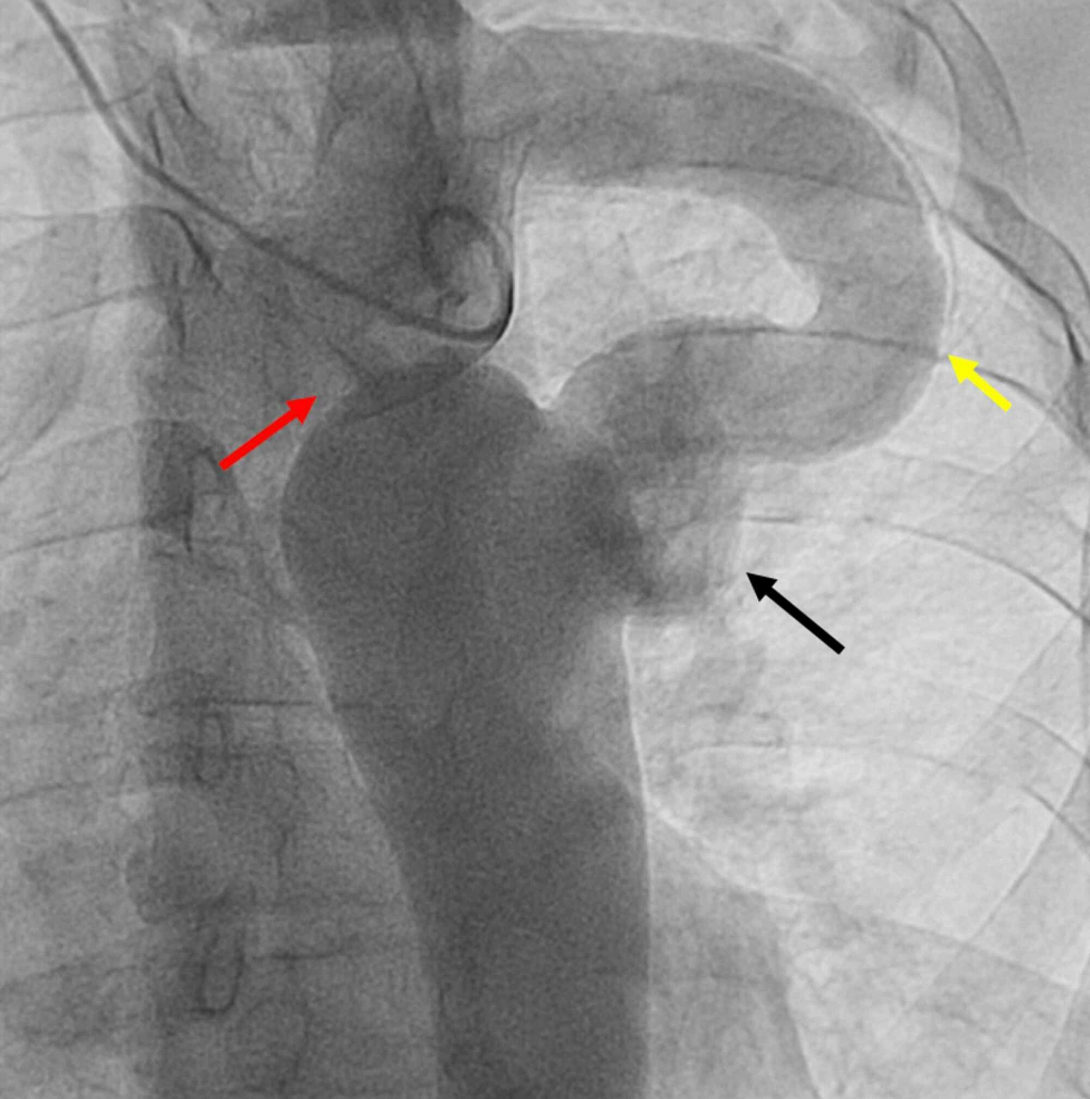
Aortic angiography revealing interrupted aorta (red arrow), extra-anatomic bypass graft from the left subclavian artery to the descending aorta (yellow arrow), and complex pseudoaneurysm at the distal anastomosis

A 52-year-old male patient underwent surgical correction of interrupted CoA at the age of 20 years using EABG from the LSCA to the descending aorta. He presented with transient sudden central chest pain with sweating. He is a known smoker for more than 30 years and hypertensive for almost 10 years, on losartan 100 mg once daily. The blood pressure (BP) was 140/90 mmHg, equal in four limbs with no brachiofemoral delay. There was no ischemic electrocardiography (ECG) changes or rise in cardiac biomarkers. His transthoracic echocardiography (TTE) revealed normal left ventricular (LV) function, mild bicuspid aortic valve (BAV) regurgitation, and dilated aortic root. The patient was taken to the cardiac catheterization laboratory and underwent coronary and aortic angiography, which revealed normal coronaries, dilated ascending aorta (3.8 cm), post-ductal aortic interruption, and patent EABG with complex pseudoaneurysm at the distal anastomosis site measuring 2x2.5 cm (Figure 1).

**Figure 2:**
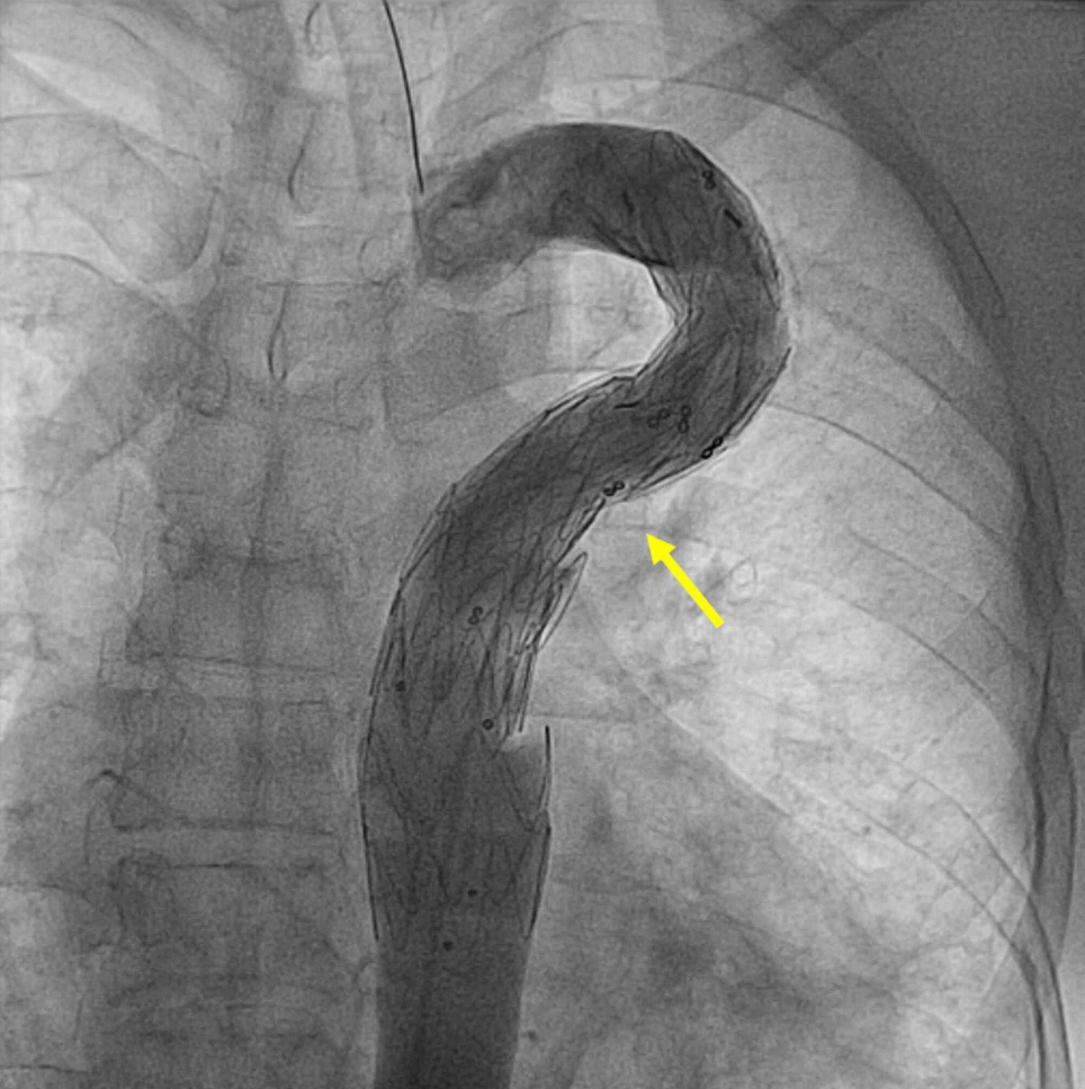
Aortic angiography revealing complete exclusion of the pseudoaneurysm after endovascular stent-graft placement (yellow arrow)

After the heart team discussion, endovascular treatment was deemed more feasible and safer than redo-surgery. We implanted two overlapped Valiant Thoracic Stent Graft (Medtronic Vascular, Santa Rosa, CA) proximally 22 mm X 100 mm and distally 28 mm X 100 mm. The aortic angiography revealed complete exclusion of the pseudoaneurysm and patent stent graft with no gradient (Figure 2).

**Figure 3:**
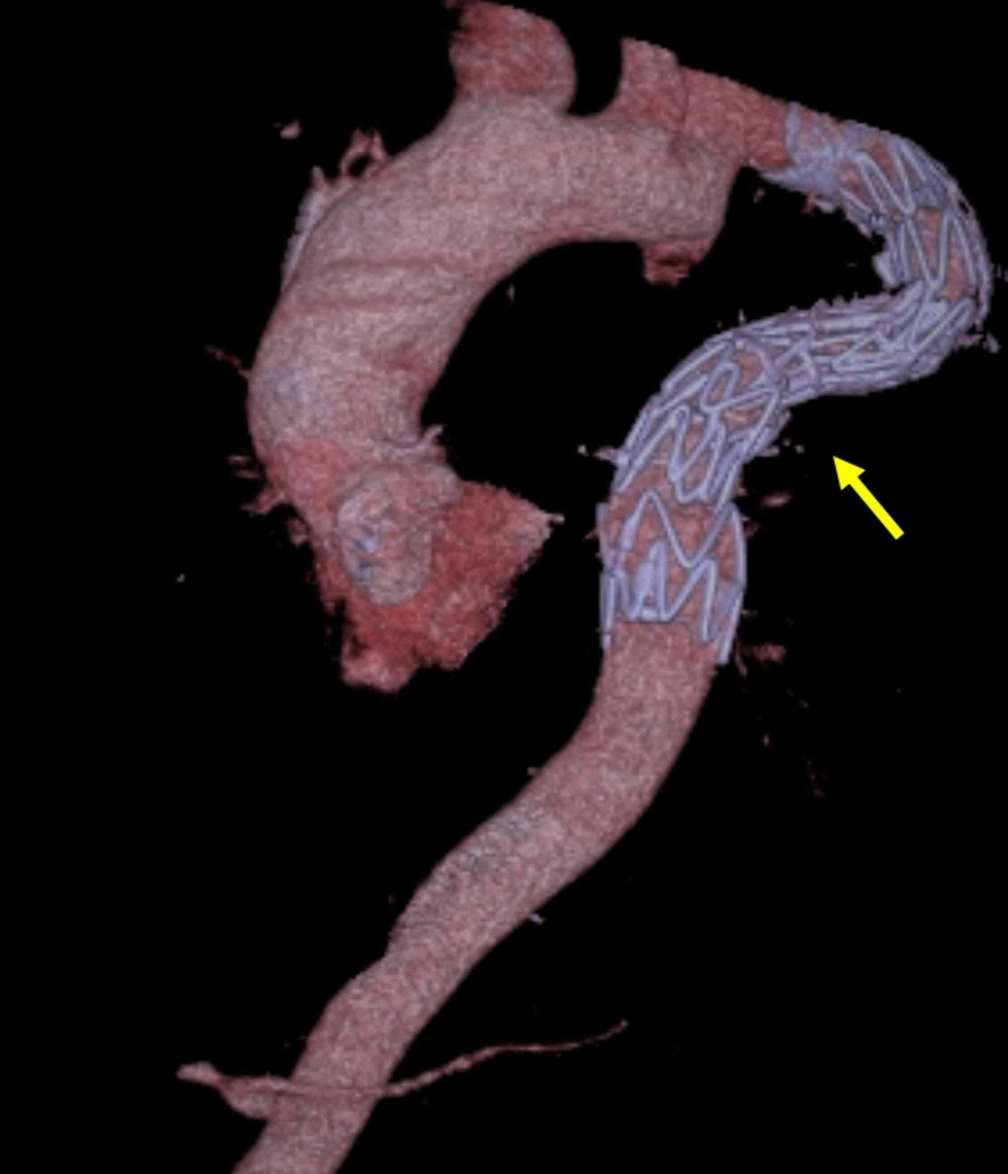
Computed tomography scan of the aorta revealing a patent stent graft with no endoleak

The patient did very well and was discharged home after two days. A follow-up computed tomographic (CT) angiography revealed a widely patent stent graft with no endoleaks (Figure 3).

Case 2

**Figure 4:**
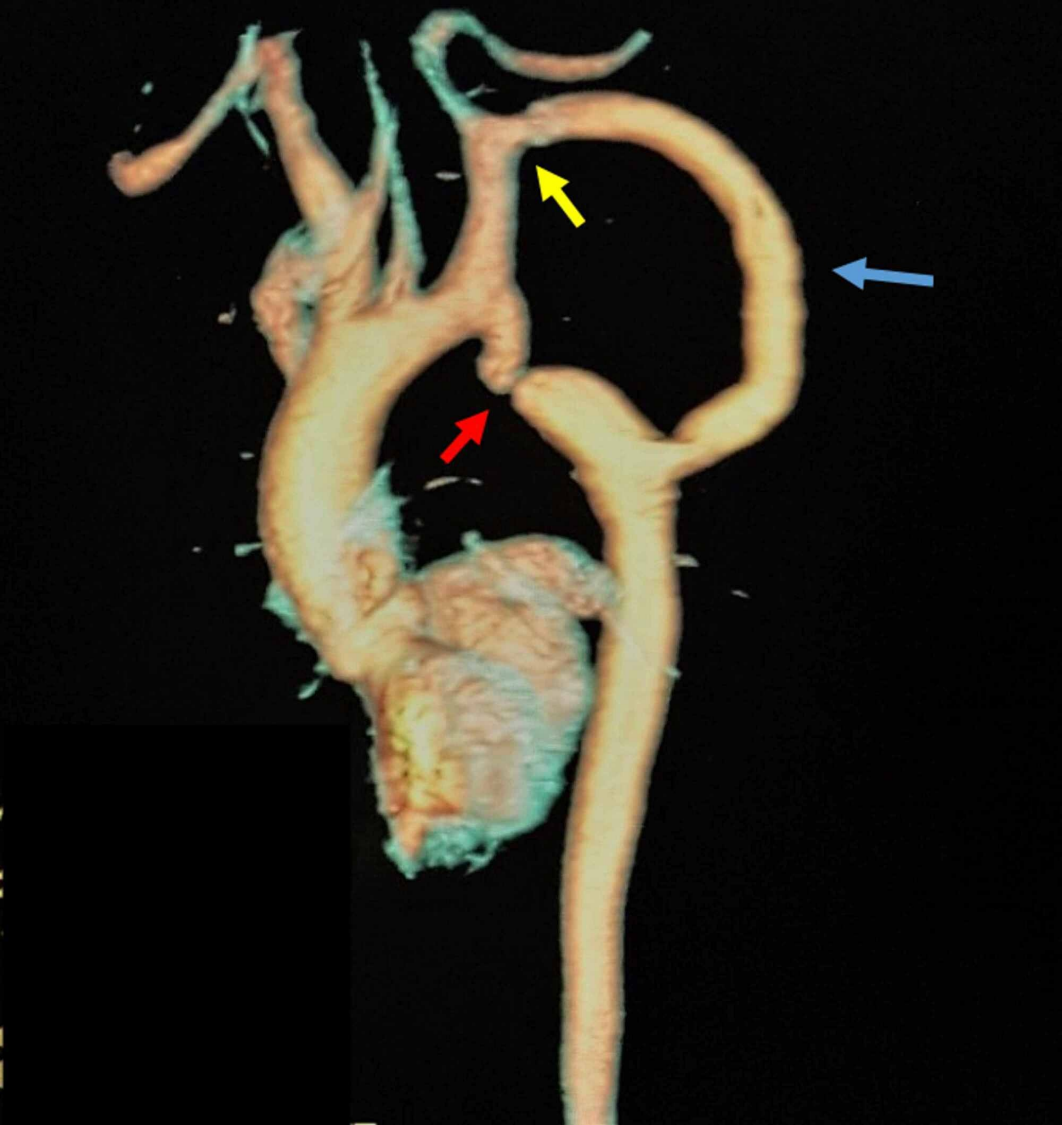
Computed tomography scan of the aorta revealing severe aortic coarctation (red arrow), extra-anatomic bypass graft from the left subclavian artery to the descending aorta (blue arrow), and stenosis at the proximal anastomosis site (yellow arrow)

A 48-year-old male patient underwent surgical correction of interrupted CoA at the age of 22 years using EABG from the LSCA to the descending aorta. He presented with shortness of breath. The patient was found to be hypertensive with a BP of 170/90 mmHg on both arms. The BP in the lower limbs was 125/85 mmHg with obvious brachiofemoral delay. The patient was taking amlodipine 10 mg and valsartan 160 mg, both once a day. The ECG revealed LV hypertrophy. His TTE revealed normal LV systolic function, with LV hypertrophy and impaired relaxation. A CT angiography revealed stenosis at the proximal anastomosis site of the EABG (Figure 4).

The plan was to proceed with cardiac catheterization and stenting the proximal stenosis after measuring the gradient but the patient refused any kind of intervention. The patient was sent home after adding a third antihypertensive medication, which wass bisoprolol 5 mg once a day. After six months, the patient's symptoms improved and his BP was 145/80 mmHg. His repeated TTE revealed no major changes.

## Discussion

EABG techniques have been accepted by most cardiac surgeons as one of the surgical approaches to repair native CoA for adult-sized patients especially when concomitant cardiac procedures need to be performed as well. Since Siderys and associates reported their first case of EABG from the ascending aorta to the abdominal aorta, distal to the renal arteries in 1974, several modifications were introduced to this technique [5]. In 1980, Vijayanagar et al. described the EABG from the ascending aorta to the descending aorta [5]. Recently, the EABG from the LSCA or axillary arteries to the descending thoracic aorta made the procedure less invasive and attractive. A study by Said et al. of 80 patients who underwent ascending-to-descending aorta EABG then followed for seven years ± six years showed significant blood pressure improvement, no early deaths, and no strokes or paraplegia [6]. However, 6% had late deaths while 4% required re-intervention for mitral valve replacement and peri-prosthetic regurgitation. Interestingly, no re-CoA and pseudoaneurysm were reported in this study, and this is probably related to the relatively short period of follow-up. However, there are several reported cases that described both re-CoA and pseudoaneurysm formation as late complications after EABG [7-9]. Such complications can occur with any of the other surgical techniques used for CoA repair with varying degrees of incidence and mandate immediate therapeutic intervention. Re-CoA and its related uncontrolled BP may lead to heart failure, premature coronary artery disease, stroke, aortic dissection, and death. Endovascular thereby for re-CoA is a well-established therapeutic modality with excellent outcomes [10-11]. If treated conservatively, pseudoaneurysms are associated with 100% rupture in one reported series [12]. Most of the reported cases of such complications were treated successfully with endovascular therapy [4,7]. Redo open surgery is challenging and associated with significant mortality and morbidity as compared to native CoA repair [4]. In our series, we demonstrated that such long-term complications can happen and continuous surveillance is mandatory for early diagnosis and treatment. Also, we demonstrated that endovascular thereby for a pseudoaneurysm is feasible and safe.

## Conclusions

On the basis of available data and this case series, long-term close follow-up evaluation is mandatory for all the patients who underwent surgical repair for CoA regardless of the type of surgery. Endovascular therapy is a less invasive alternative to surgery for patients who have undergone previous aortic repairs and presented with re-CoA and/or pseudoaneurysm.
